# Anti-Colitic Effect of an Exopolysaccharide Fraction from *Pediococcus pentosaceus* KFT-18 on Dextran Sulfate Sodium-Induced Colitis through Suppression of Inflammatory Mediators

**DOI:** 10.3390/polym14173594

**Published:** 2022-08-31

**Authors:** Jeong-Hun Lee, Kyung-Sook Chung, Ji-Sun Shin, Seang-Hwan Jung, Sangmin Lee, Myung-Ki Lee, Hee-Do Hong, Young Kyoung Rhee, Kyung-Tae Lee

**Affiliations:** 1 Department of Pharmaceutical Biochemistry, College of Pharmacy, Kyung Hee University, Seoul 02447, Korea; 2 Department of Biomedical and Pharmaceutical Sciences, Graduate School, Kyung Hee University, Seoul 02447, Korea; 3 Department of Pharmacy, College of Pharmacy, Kyung Hee University, Seoul 02447, Korea; 4 Korea Food Research Institute, Wanju-gun 55365, Korea

**Keywords:** exopolysaccharide, *Pediococcus pentosaceus*, DSS-induced colitis, NF-κB, STAT1

## Abstract

We previously reported the immunostimulatory effect of an exopolysaccharide fraction from *Pediococcus pentosaceus* KFT18 (PE-EPS), a lactic acid bacterium, in macrophages and primary splenocytes, as well as in cyclophosphamide-induced immunosuppressed mice. In this study, the anti-colitic activity of PE-EPS was investigated in a dextran sulfate sodium (DSS)-induced colitis animal model. PE-EPS relieved DSS-induced colitis symptoms, such as stool blood, decreased colon length, crypt disruption, and mucus layer edema. Regarding the molecular mechanism, PE-EPS reduced the enhanced expression of inducible nitric oxide synthase (iNOS), cyclooxygenase-2 (COX-2), and pro-inflammatory cytokines (TNF-α, IL-6, and IL-1) in the colon tissue of colitis-induced mice. Additionally, PE-EPS protected against DSS-induced phosphorylation of p65 and signal transducer and activator of transcription 1 (STAT1). These findings suggested that the exopolysaccharide fraction from *Ped. pentosaceus* KFT18 can be used to treat inflammatory bowel disease by alleviating colonic inflammation.

## 1. Introduction

Inflammatory bowel diseases (IBD), including ulcerative colitis or Crohn’s disease, are chronic inflammatory disorders of the gastrointestinal system [1]. Accordingly, IBD has been investigated to understand the mechanisms of the disease’s progression and to identify therapeutic targets. IBD is thought to be caused by several factors, including poor eating habits and immunological responses [2]. Symptoms (weight loss, abdominal pain, diarrhea, and bloody stool) and inflammatory responses (influx of neutrophils and macrophages that secrete cytokines) in the intestinal mucosa are characteristic of IBD [3].

It has been previously reported that the development of IBD is highly linked to pro-inflammatory cytokines such as interleukin (IL)-1ꞵ, (IL)-6, and tumor necrosis factor-alpha (TNF-α) [4,5,6]. Therefore, antibodies that neutralize pro-inflammatory cytokines have been considered for the treatment of IBD for in vivo [7] or clinical trials [8]. Several studies have suggested a variety of biological factors (anti-TNF-α, anti-integrins, or anti-ILs) and small molecules (tofacitinib, a non-selective Janus kinase (JAK) inhibitor) as the therapeutic targets for the treatment of IBD. However, as those products have safety issues involving headaches, dizziness, dermatologic problems, and acute anaphylaxis [9,10,11], further studies on the development of medicines and healthy functional foods for IBD are required.

Nuclear factor-kappa B (NF-κB) regulates both innate and adaptive immune responses and is transiently induced by oxidative stress or pro-inflammatory cytokines [12]. A variety of stimuli including ligands of cytokine receptors, TNF receptors, or pattern-recognition receptors initiate the canonical NF-κB pathway and induce transient nuclear translocation of p50/RelA or p50/c-Rel dimers, which activate inducible nitric oxide synthase (iNOS), cyclooxygenase-2 (COX-2), and pro-inflammatory cytokines [13,14]. Therefore, it is assumed that the NF-κB pathway is the potential target as an anti-colitic agent [15,16].

Signal transducer and activator of transcription 1 (STAT1) is a transcriptional regulator involved in the pathogenesis of the inflammatory disease. Translocation, DNA-binding activity, and gene expression of STAT1 have been observed in the colonic tissue of IBD patients [17,18]. Therefore, targeting this transcription factor has been frequently adopted as a therapeutic strategy for IBD. For example, tofacitinib a JAK inhibitor has been developed as a medicine for ulcerative colitis through the JAK-STAT pathway inhibition and has been recently approved by the FDA for ulcerative colitis [19]. Additionally, natural products to inhibit the JAK-STAT pathway have been investigated to treat ulcerative colitis [20].

*Ped. pentosaceus* contains metabolites, such as lactic acid bacteria and bacteriocin, which make it useful in food preservation. Furthermore, numerous strains of *Ped*. *pentosaceus* have been shown to have probiotic effects by themselves [21]. Kimchi, a typical Korean fermented meal, was used to isolate *Ped. pentosaceus* KFT-18 [22]. We previously reported that PE-EPS, an exopolysaccharide fraction extracted from *Ped*. *Pentosaceus* KFT18 can increase NO and cytokines production pathway in the IFN-activated macrophages via upregulation of the NF-κB pathway and IL-2 and IFN-γ productions in the anti-CD3/CD28-co-primed primary splenocytes [23]. PE-EPS has been reported to show immunostimulatory effects in the cyclophosphamide (CYP)-induced immunosuppressed mouse models, recovering thymus and spleen conditions and hematological parameters such as the lymphocyte and neutrophil count. Nevertheless, the anti-colitic action of PE-EPS in inflammatory conditions is yet to be elucidated. In this regard, we hypothesized that the immunostimulatory effect of PE-EPS can be associated with the homeostasis of the intestinal epithelium in experimental colitis and contribute to the amelioration of colitis development. In this study, we aimed to evaluate the anti-colitic effect and the molecular mechanism of PE-EPS in the dextran sodium sulfate (DSS)-provoked colitis mouse model.

## 2. Materials and Methods

### 2.1. Chemicals and Reagents

All reagents and antibodies used in experiments were described in Supplementary Materials (Supplementary Tables S1 and S2)

### 2.2. PE-EPS Preparation

PE-EPS exopolysaccharides were prepared as described in our previous work [23]. Briefly, the strain used in this experiment was *Ped. Pentosaceus* KFT18 (KCCM No. KCCM11309P) isolated from Kimchi. After incubation of *Ped. Pentosaceus* KFT18 with Man Rogosa and Sharpe broth (Difco Laboratories, Detroit, MI, USA) with 2% sucrose for 48 h, the supernatant was precipitated with ethanol, and PE-EPS was obtained. Proteins (3.5%*w*/*w*) and carbohydrates (96.4%) were identified in the isolated PE-EPS, with the carbohydrate component having a mixture of acidic (37.9%) and neutral (58.5%) sugars. PE-EPS contains more than 63% high molecular weight polysaccharides (>2560 KDa), and its components have been described in our previous work [23].

### 2.3. DSS-Induced Colitis Mouse

**Figure 1:**
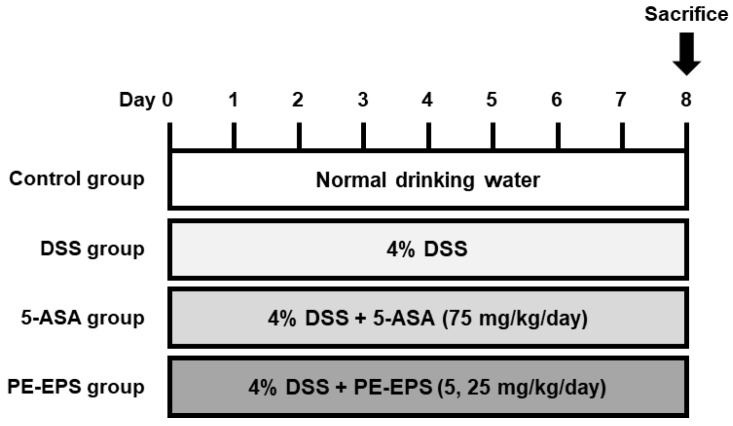
Administration scheme of the DSS-induced colitis mouse model. Mice were provided with 4% DSS in drinking water (ad libitum feeding) for seven days, with or without PE-EPS (5 or 25 mg/kg/day, p.o.). The positive control entailed 5-ASA (75 mg/kg/day, p.o.).

Seven-week-old male ICR mice were obtained from ORIENT BIO Inc. (Gyeonggi-do, Republic of Korea). For one week, all mice were acclimated and fed rodent standard laboratory chow on a 12 h light–dark cycle under constant conditions (temperature, 20 ± 5 °C; humidity, 40–60%). The IBD mouse model was established after 7 day-acclimation with drinking water containing 4% DSS (*w*/*v*) for seven days. The mice were categorized into five groups (*n* = 6 per group): (1) vehicle control group (mice drinking pure water and taking a daily vehicle per oral [p.o.]); (2) DSS group (mice drinking water with DSS and taking a daily vehicle [p.o.]); (3) 5-ASA group (mice drinking water containing DSS and taking daily 5-ASA [75 mg/kg, p.o.]); (4) and (5) PE-EPS group (mice drinking water containing DSS and taking daily PE-EPS [5 or 25 mg/kg, p.o.]). Before treatment, PE-EPS and ASA were dissolved in the vehicle (0.9% saline) and orally administrated as a solution state. Figure 1 shows the administration schedule for each reagent.

During treatment, the disease activity index (DAI) was evaluated via daily recordings of weight loss, stool consistency, and hemoccult (Table 1). Each score was made as follows: body weight loss (0: none, 1: 1–5%, 2: 5–10%, 3: 10–20%, 4: >20%), stool consistency (0: normal, 1 and 2: loose stool, 3 and 4: diarrhea), and hemoccult (0 = no blood, 2 = blood trace in the stool clearly visible, 4 = gross rectal bleeding). On day 7, the mice were sacrificed, and their colons were removed from the proximal rectum, close to their passage under the pelvisternum. Their colon lengths were measured between their ileocecal junction and proximal rectum.

### 2.4. Hematoxylin and Eosin (H&E) Staining

Colon tissues were embedded in paraffin after treatment with 4% paraformaldehyde in phosphate-buffered saline (PBS). H&E staining was conducted by Korea Experimental Pathology, Inc. (Gyeonggi-do, Republic of Korea). The stained slides were studied and photographed using a microscope (OLYMPUS cellSens Standard 1.9., Tokyo, Japan).

### 2.5. Protein Extraction and Western Blot Analysis

Colon tissues were obtained and homogenized using Tissue-Tearor^TM^ (BioSpec Products Inc., Bartlesville, OK, USA) with PRO-PREPTM protein lysis buffer (Intron Biotechnology, Seongnam, Korea). After incubation at 4 °C for 30 min, the mixtures were centrifuged (4 °C, 10,000× *g* for 40 min), and the supernatants were collected. A Bradford assay was performed using a reagent (Bio-Rad, Hercules, CA, USA) to measure protein concentration. The equivalent amounts of protein were separated using 8–15% SDS-PAGE, and then transferred to polyvinylidene difluoride (PVDF) membranes. The membranes were incubated with non-fat dry milk containing the primary antibodies at 4 °C overnight. After incubation, the membranes were washed three times with 0.1% Tween-20-containing Tris-buffered saline (T-TBS). They were incubated with the secondary antibodies conjugated with horseradish peroxidase (HRP) for 2–4 h at room temperature (18–22 °C). The membranes were washed with T-TBS three times and developed with an enhanced chemiluminescence detection system (Amersham, Little Chalfont, UK).

### 2.6. Quantitative Real-Time Reverse-Transcriptase Polymerase Chain Reaction (qRT-PCR)

Easy Blue^®^ kits (Intron Biotechnology, Seoul, Korea) were used to extract cellular RNA. TOPscript™ RT Dry MIX was used to reverse-transcribe an equivalent amount of RNA (500 ng per sample), and a Thermal Cycler Dice Real-Time System (TaKaRa Bio Inc., Shiga, Japan) was used to perform quantitative real-time PCR amplification. The oligonucleotide primers which are listed in Supplementary Table S3 are designed by Primer3 and the specificity checking module uses BLAST. Amplification was measured by incorporation of TB Green^TM^ Premix Ex Taq (TaKaRa Bio Inc., Shiga, Japan) to detect TNF-α, IL-1β, IL-6, and β-actin mRNA expression. PCRs were carried out for 50 cycles applying the following conditions: denaturation at 95 °C for 5 s, annealing at 55 °C for 10 s, and elongation at 72 °C for 20 s. The mean Ct value of the gene of interest was calculated using triplicate measurements and normalized against the mean Ct value of the control gene, β-actin.

### 2.7. Statistical Analysis

GraphPad Prism^®^ version 8.0.1 software (GraphPad Software Inc., La Jolla, CA, USA) was used to conduct the statistical analysis. Data are described as the mean ± standard error (SEM). One-way analysis of variance (ANOVA) and Bonferroni’s post hoc test were used to examine statistical differences, with *p* < 0.05 being considered statistically significant.

## 3. Results

### 3.1. PE-EPS Restores Clinical Symptoms in DSS-Induced Colitis Mice

**Figure 2:**
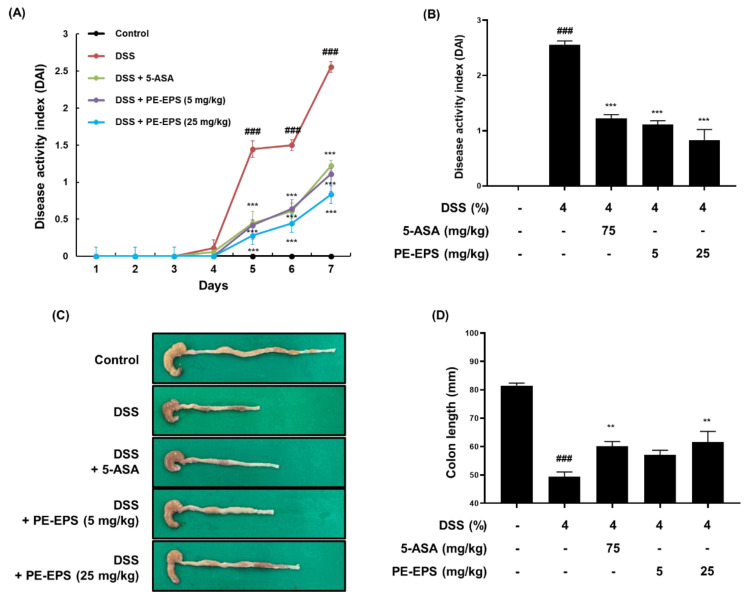
The anti-colitic effects of PE-EPS on the DSS-induced colitis model. (**A**,**B**) All mice except for the control group were fed 4% DSS in their drinking water, and co-treated with 5-ASA (75 mg/kg/day, p.o.) or PE-EPS (5 or 25 mg/kg/day) for 7 days. The disease activity index (DAI) score was measured through observation of pathological symptoms such as loss of body weight, stool consistency, and gross bleeding. (**C**,**D**) The colon tissue was extracted and measured with a caliper, and the colon length in each group is indicated in the graph. Values are the mean ± SEM (*n* = 6); ^###^ *p* < 0.001 vs. the vehicle control group; ** *p* < 0.01, *** *p* < 0.001 vs. the DSS group.

We employed a DSS-induced colitis mouse, which has been widely used for its similarities to the human IBD model [27], and assessed the DAI score daily based on pathological symptoms like stool blood, stool consistency, and body weight change. We found that the DAI score of the DSS group significantly increased (DAI score: 2.56 ± 0.38), but that the score of the 5-ASA group (DAI score: 1.22 ± 0.46) and PE-EPS group (DAI score: 1.20 ± 0.36 and 0.90 ± 0.48 after treatment with 5 and 25 mg/kg/day PE-EPS, respectively) attenuated the elevated DAI score by DSS administration (Figure 2A,B). After treatment, we assessed the colon length derived from the mice as an evaluation factor for the occurrence of colitis. As shown in Figure 2C,D, 4% DSS induced a decrease in colon length compared to the vehicle control group. However, 5-ASA (75 mg/kg/day) or PE-EPS (5 or 25 mg/kg/day) treatment potently restored the colon length.

**Figure 3:**
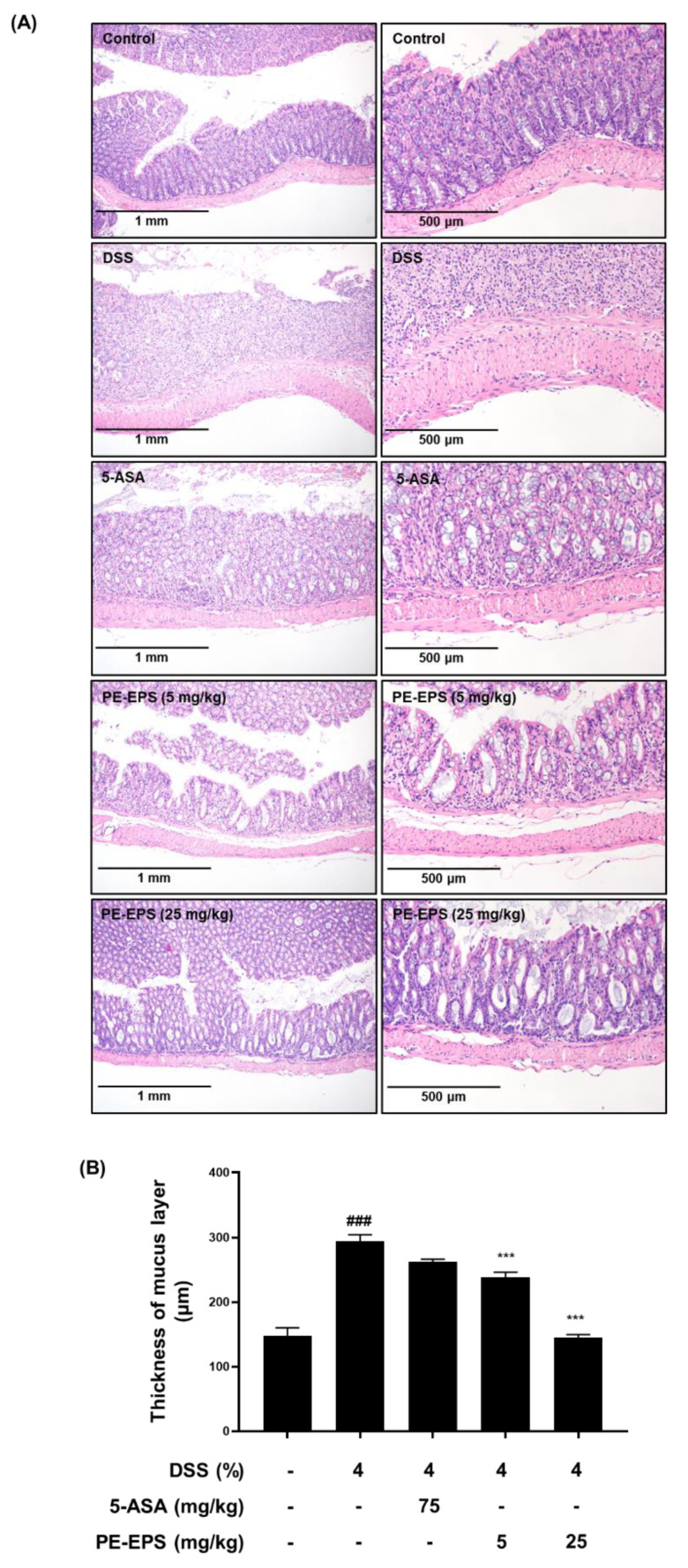
Histopathological effects of PE-EPS on the colon tissues of the DSS-induced colitis model. (**A**) Representative sections of the colon tissues from the mice fed 4% DSS in drinking water for 7 days with PE-EPS (5 or 20 mg/kg/day, p.o.). 5-ASA (75 mg/kg/day, p.o.) was prepared as a positive control. The colon tissues were isolated from mice and then fixed with 4% formaldehyde in PBS. The fixed tissues were stained with H&E staining and observed with the hallmarks of colitis by a microscope. (**B**) The thickness of the mucus layer was measured by the Olympus program. Values are the mean ± SEM (*n* = 6); ^###^ *p* < 0.001 vs. the vehicle control group; *** *p* < 0.001 vs. the DSS group.

Furthermore, isolated colon tissues were stained with H&E, and their pathological alterations were analyzed. Disruption of crypts and enlargement of the submucosal layer in DSS-treated colon tissue have been reported as hallmarks of the colitis state [28]. Compared to the vehicle control group, the DSS-treated mice showed villus loss, disruption of crypts, and edema of the muscle layer, meaning an expansion between the mucosa and submucosa (Figure 3A). The shape of the colon tissue and symptoms were significantly (*p* < 0.001) recovered in the PE-EPS (5 or 25 mg/kg/day) or 5-ASA-treated groups. The muscle layer of colon tissue had become thicker in DSS-induced mice, while treatment of PE-EPS (5 or 25 mg/kg/day) or 5-ASA restored these symptoms (Figure 3B). These findings imply that oral administration of PE-EPS protects against DSS-induced colonic damage.

### 3.2. PE-EPS Decreases the mRNA and Protein Levels of iNOS and COX-2 in the DSS-Induced Colitis Mice

**Figure 4:**
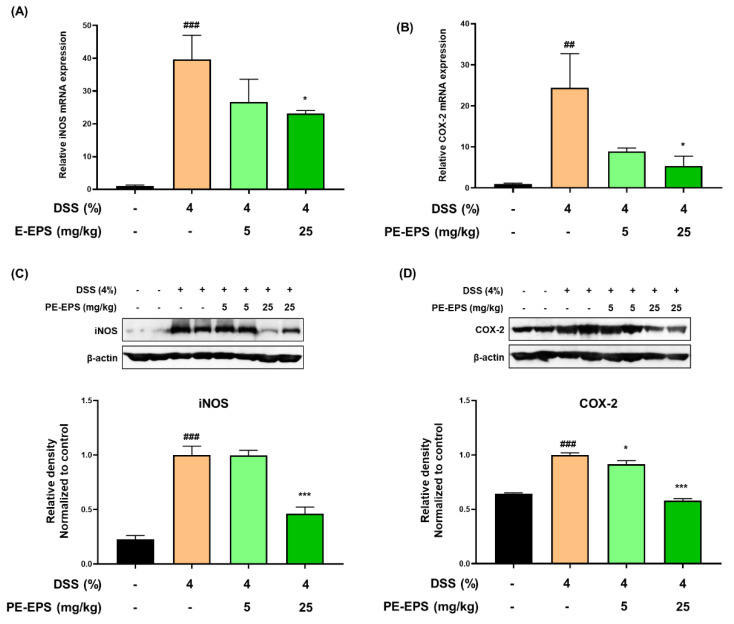
The effects of PE-EPS on the expression levels of iNOS and COX-2 mRNA and protein of the DSS-induced colitis model. (**A**,**B**) The colon tissues were homogenized and purified RNA was obtained. The mRNA expression level of iNOS or COX-2 was measured by qRT-PCR. The expression levels of iNOS and COX-2 were adjusted with β-actin. (**C**,**D**) The colon tissues were homogenized in a protein lysis buffer, and the protein lysates were separated by SDS-PAGE, and then transferred to the PVDF membranes. The antibodies against iNOS and COX-2 were used to detect protein levels of iNOS and COX-2, and β-actin was an internal control. The relative densitometry of blot data was measured by Quantity one. Values are the mean ± SEM (*n* = 6); ^##^ *p* < 0.01, ^###^ *p* < 0.001 vs. the vehicle control group; * *p* < 0.05, *** *p* < 0.001 vs. the DSS group.

When the tissues are damaged by colitis, the pro-inflammatory proteins such as iNOS and COX-2 are upregulated in the colon tissue, and their expression levels are related to the colonic damage [29]. We analyzed iNOS and COX-2 mRNA expression levels in the colon tissue. As shown in Figure 4A,B, DSS treatment markedly increased mRNA levels of iNOS and COX-2 in the colon tissue, whereas PE-EPS treatment, especially at a high dose (25 mg/kg/day), significantly lowered the mRNA levels of iNOS and COX-2. Next, we performed a Western blotting assay to investigate whether PE-EPS affected iNOS and COX-2 protein expression. Protein expression levels of iNOS and COX-2 were significantly higher in the DSS group than ones in the vehicle control group (Figure 4C,D). However, PE-EPS treatment potently suppressed iNOS and COX-2 expressions in the colon tissues of the DSS-treated mice. These results importantly suggest that PE-EPS may block the DSS-provoked inflammation through the reduction of pro-inflammatory mediators.

### 3.3. PE-EPS Suppresses Production of Pro-Inflammatory Cytokines of the DSS-Induced Colitis Mice

**Figure 5:**
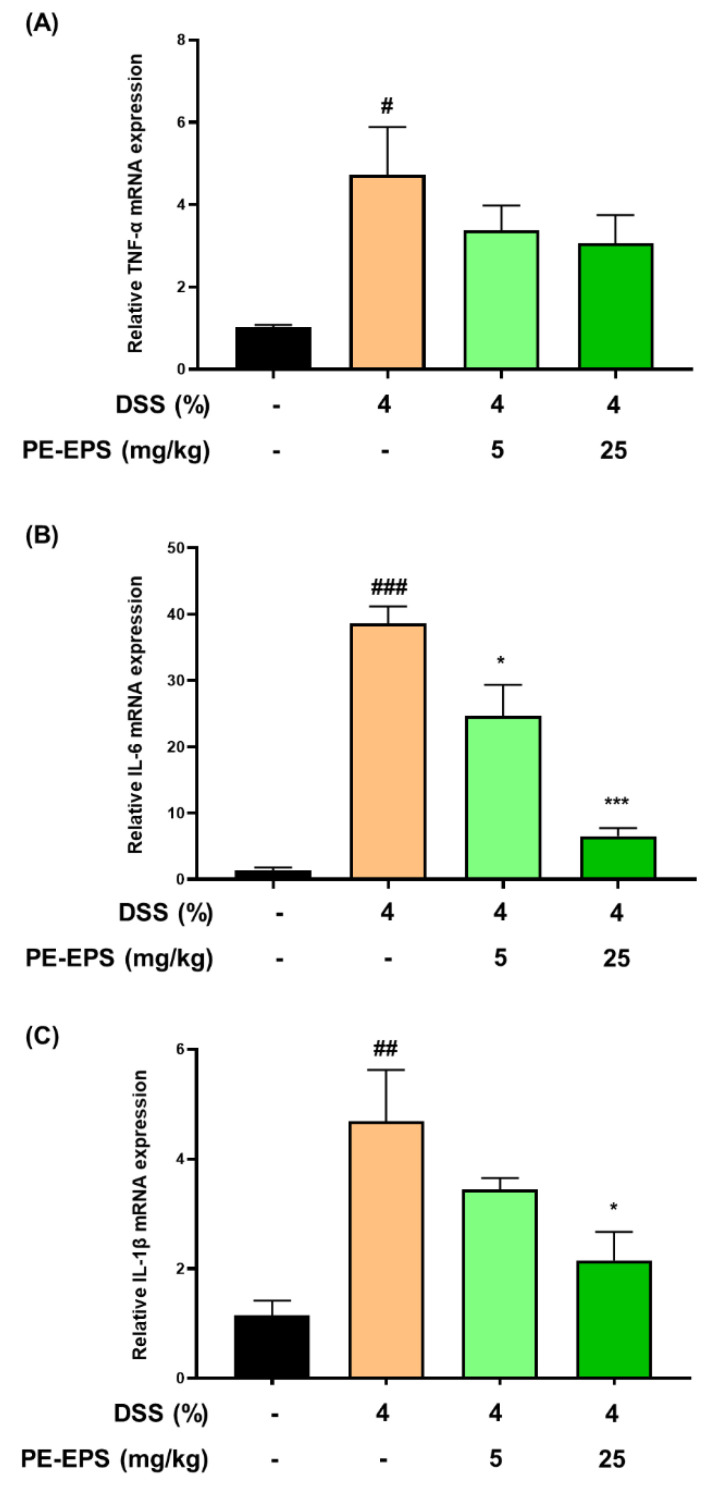
The effect of PE-EPS on mRNA expression levels of the pro-inflammatory cytokines in the DSS-induced colitis model. (**A**–**C**) The colon tissues were homogenized, and purified RNA was obtained. The mRNA expression levels of TNF-α, IL-6, and IL-1β were quantified by qRT-PCR. The levels of TNF-α*,* IL-6, and IL-1β were adjusted by β-actin. Values are the mean ± SEM (*n* = 6); ^#^ *p* < 0.05, ^##^ *p* < 0.01, ^###^ *p* < 0.001 vs. the vehicle control group; * *p* < 0.05, *** *p* < 0.001 vs. the DSS group.

In the colon tissue or blood from DSS-induced colitis mice, the mRNA and protein levels of pro-inflammatory cytokines (TNF-α, IL-6, and IL-1 β) are enhanced [30,31,32]. Based on the reports, we thereby evaluated the mRNA expression levels of TNF-α, IL-6, and IL-1β in colonic tissue. The mRNA expression levels of TNF-α, IL-6, and IL-1β in colon tissue of the DSS-induced colitis group were considerably elevated by the 4% DSS treatment (Figure 5A–C). IL-6 and IL-1β mRNA expression levels were significantly suppressed in colon tissue after the PE-EPS treatment, particularly in the high-dose (25 mg/kg/day)-treated mice, but TNF-α was not significantly affected. These findings imply that PE-EPS treatment can reduce the expression of pro-inflammatory cytokines such as IL-1β and IL-6 in the colitis colon tissues.

### 3.4. PE-EPS Suppresses DSS-Induced Activation of the NF-κB and STAT1 Pathways in Colons of the Colitis Mice

**Figure 6:**
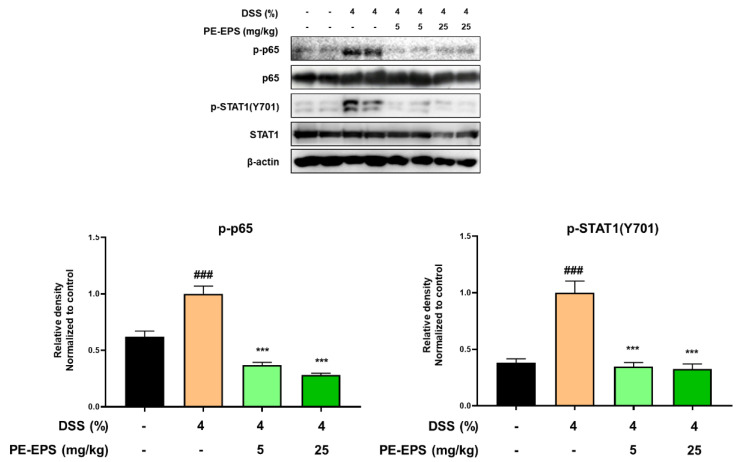
Effect of PE-EPS on phosphorylation levels of p65 and STAT1 in the DSS-induced colitis model. The colon tissues were homogenized in protein lysis buffer, and protein lysates were separated by SDS-PAGE, and then transferred to the PVDF membrane. The protein expression level was measured using antibodies against phospho-p65, p65, phospho-STAT1, and STAT1. As an internal standard, β-actin was used. The densitometry of blot data was measured by Quantity one. Values are the mean ± SEM (*n* = 6); ^###^ *p* < 0.001 vs. the vehicle control group; *** *p* < 0.001 vs. the DSS group.

In ulcerative colitis, NF-κB and STAT1 are frequently studied as important transcriptional factors that can control the expression of cytokines, and provoke immunological responses including inflammation in the intestinal tract [33,34]. Therefore, we studied the effects of PE-EPS on phosphorylation levels of p65 and STAT1 in the colon tissues of the DSS-treated mice by a Western blot analysis. The colon tissue extracted from the DSS-treated group confirmed the enhanced phosphorylation levels of p65 and STAT1, but the samples from the PE-EPS-treated mice were similar to the vehicle control group (Figure 6). These findings indicate that suppression of iNOS, COX-2, and pro-inflammatory cytokines are responsible for the anti-colitic effects of PE-EPS.

## 4. Discussion

EPS from bacteria is a surface molecule that acts as a crosstalk mediator between probiotics and the host. The stimulation of macrophages by EPS from lactic acid bacteria revealed their significant immunomodulatory and anti-tumor properties [35]. In previous reports, treatment of intestinal epithelial cells with EPS from *Lactobacillus reuteri* promoted upregulation of the pro-inflammatory factors (TNF-α, IL-6, and NF-κB) and inhibited adherent contacts with some gut bacteria such as *Escherichia coli* [36]. Liu et al. demonstrated that exopolysaccharides from *Lactobacillus helveticus* relieved symptoms of the DSS-induced colitis mice model [37]. EPS produced by *Streptococcus thermophilus* protected intestinal barrier integrity from the disruption by lipopolysaccharide in Caco-2 monolayer, increased expression of the tight junction and alleviated pro-inflammatory response [38]. In addition, we reported that EPS from *Bacillus subtilis* with proven immune enhancement contributed to the maintenance of the intestinal barrier integrity in the DSS-induced colitis mouse model via regulation of the immune cells and inflammatory response [24]. Based on several previous studies, we speculated on the development possibility of EPS as a therapeutic agent for IBD. Therefore, in the present study, we investigated the anti-colitis effects of the bacterial EPS extracted from *Pediococcus pentosaceus* KFT-18 in the DSS-induced colitis mice to alleviate the immune responses in the IBD.

We used several evaluation factors to quantify the induction of colitis in an IBD mouse model. DAI was performed, and the entire colon length was measured because drinking water with DSS causes acute colitis with recognizable symptoms, such as hyperemia and bloody diarrhea [39]. PE-EPS treatment significantly reduced elevated DAI scores and intestinal contractions caused by DSS administration. H&E staining was also used to assess histopathological changes such as granulocyte infiltration and disruption in the basal crypts of intestinal epithelial cells [40,41]. The crypt is a tube-like gland in the colon, and pluripotent stem cells are located at the bottom of the crypt. Stem cells can reproduce and renew the colonic epithelium to provide tissue homeostasis and mucosal barrier integrity [42]. Our data revealed that the DSS treatment induced a crypt collapse and submucosal edema, but PE-EPS treatment significantly reversed the histological damages, supposing that PE-EPS might regulate the expression of tight junction protein. Tight junctions are multiple protein complexes composed of four transmembrane proteins, such as claudins, occludin, junctional adhesion molecules, tricellulin, and a wide spectrum of cytosolic proteins involving zonulae occluden and cingulin [43]. The downregulation of tight junction barrier function is closely associated with the invasion of intestinal pathogens in the colon wall leading to the pathogenesis of intestinal inflammation.

Infection or injury triggers an inflammatory response, causing overexpression of various pro-inflammatory factors, such as iNOS, which produces nitric oxide throughout the inflammatory responses. COX-2 is also upregulated during the inflammatory response and produces prostaglandins, a bioactive molecule derived from arachidonic acid [44]. As these enzymes are important in the modulation of inflammation, we examined their mRNA and protein levels. We found out that PE-EPS suppressed the DSS-elevated levels of iNOS and COX-2 mRNAs and proteins. These findings were consistent with previous reports [25,45], which indicated that the PE-EPS treatment reduced inflammatory responses in the colon tissues. Among the inflammatory mediators, cytokines are functional proteins secreted by various cells for cell interactions and communication [46]. Pro-inflammatory cytokines (TNF-α, IL-6, and IL-1β) and anti-inflammatory cytokines (IL-4, IL-10, IL-11, and IL-13) are examples of cytokines that regulate inflammatory responses by activating or suppressing immune cells [46]. These pro-inflammatory cytokines are particularly abundant in immune cells (macrophages, monocytes, T cells, and B cells) and play important roles in inflammatory diseases [47]. We used qRT-PCR to examine the mRNA expression levels of pro-inflammatory cytokines, including TNF-α, IL-6, and IL-1β, in colon tissue because higher protein and mRNA levels of these cytokines were observed in the DSS-induced colitis animal model [48,49]. The results showed a potent decrease in IL-6 and IL-1β mRNA levels following the PE-EPS treatment. Therefore, further studies on other factors that may affect DSS-induced colitis, such as anti-inflammatory cytokines or tight junction proteins are needed.

Cytokine-induced transcription factor activation may reveal additional insights into key steps of the complex cytokine network in IBD therapy [17]. In the mucosal immune system, many immune regulatory genes contain specific binding sites for the STAT family and NF-κB in their promoter regions. STAT proteins are dormant cytoplasmic transcription factors that become activated after phosphorylated by Janus kinases (JAK) or other kinases in response to the binding of cytokine or growth factor receptors. STAT homo- or STAT heterodimers indicating activation translocates into the nucleus and binds to specific promoter elements to regulate inflammatory gene expression. Among the STAT family, since the activation of STAT1 triggers an important signaling pathway for many cytokines, various pro-inflammatory proteins such as iNOS and COX-2, and growth factor receptors [50], the STAT1 signaling pathway might play a role in the pathogenesis of IBD. Similar to the STAT family, NF-κB also plays an essential role in the signaling of inflammatory reactions that produce a variety of cytokines and chemokines [13]. NF-κB-induced cytokines further stimulate, activate and differentiate lamina propria immune cells, which aggravate the perpetuation of mucosal inflammation [16]. In these regards, since these transcriptional factors contribute to the development of IBD [51,52,53], we examined the phosphorylation levels of STAT1 and p65, which are involved in the activation of the STAT1 and NF-κB pathways. In the present study, phosphorylation of p65 and STAT1 was significantly reduced by the anti-colitis effects of PE-EPS in the DSS-induced colitis mice, and we speculated a prevention possibility of signaling crosstalk between STAT1 and NF-κB by PE-EPS treatment. The phosphorylation and nuclear translocation of STAT1 homodimers upregulate the expression of several signaling components of the TLR signaling pathway, such as TLRs and co-receptors MD2 and CD14 [54]. SOCS1, a negative regulator of STAT1 signaling, promotes the degradation of TIRAP, which recruits MyD88 to TLR2 and TLR4 triggering NF-κB transcription. Consequently, we recognized that STAT1 and NF-κB pathways can be a key target of PE-EPS for IBD, and at the same time considered that further work will be required to delineate the regulation of crosstalk between STAT1 and NF-κB in various molecular biological analysis.

In the present study, we realized many limitations in our research to prove the improvement mechanism of PE-EPS in IBD. It has been shown that polysaccharides can play direct and indirect roles in efficacy with multichannel, multilevel, and multitarget processes [55]. Thereby, at this stage, it is even more difficult to estimate whether PE-EPS or its metabolites are pharmacodynamic active substances, especially after oral administration. Nevertheless, their biological activities are closely correlated to their chemicophysical properties including their structure, molecular weight, and charge through the pharmacokinetic or pharmacodynamic pathways [56]. To overcome the limitations of our research, we are preparing the pilot scale production of PE-EPS with Korea Food Research Institute and will further investigate the detailed molecular mechanisms including intestinal barrier integrity via regulation of tight junction protein, modulation of immune cell-related inflammatory mediators, the crosstalk signaling between transcription factors, and transition of microbiota composition and the pharmacokinetic pathway to develop the future therapeutic agents using PE-EPS in IBD.

## 5. Conclusions

In summary, PE-EPS treatment attenuates the inflammatory response such as iNOS, COX-2, and other pro-inflammatory cytokines expressions in the colonic tissues by suppressing the STAT1/NF-κB pathway, resulting in the repair of colonic damage induced by DSS administration. The present study elucidated the mechanism underlying the anti-colitis effects of PE-EPS in the DSS-induced mice model and proposed the use of EPS generated by the probiotics in the anti-inflammatory therapy for IBD patients.

## Figures and Tables

**Table 1:** Evaluation criterion of disease activity index (DAI) [24,25,26].

Score	Weight Loss (%)	Stool Consistency	Hemoccult
0	None	Well form stool	Negative
1	1–5	-	-
2	5–10	Loose stool	Positive
3	10–20	-	-
4	>20	Diarrhea	Gross bleeding

## Data Availability

Not applicable.

## Supplementary Materials

The following supporting information can be downloaded at: https://www.mdpi.com/article/10.3390/polym14173594/s1, Table S1: Agents’ information used in experiments; Table S2: Antibody information used in experiments; Table S3: List of primers.
