# Supplementary material for: Anti-Colitic Effect of an Exopolysaccharide Fraction from Pediococcus pentosaceus KFT-18 on Dextran Sulfate Sodium-Induced Colitis through Suppression of Inflammatory Mediators
Source: Polymers (Basel). 2022 Aug 31;14(17):3594. doi: 10.3390/polym14173594 (PMC9460603; doi:10.3390/polym14173594)
Supplement: Supplementary file 1 [file polymers-14-03594-s001.zip › polymers-1798737-supplementary.pdf]

Supplementary materials

Table S1. Agents' information used in experiments.

Reagent	Source	Cat. No.
Acrylamide/bisacrylamide	Bio-Rad Laboratories	#1610158
Ammonium persulfate	(Hercules, CA, USA)	#1610700
Phenylmethylsulfonyl fluoride (PMSF)	Sigma-Aldrich (St. Louis, MO, USA)	P7626
N, N, N´, N´-tetramethylethylenediamine (TEMED)		T9281
Trizma base		T1503
Sodium dodecyl sulfate (SDS)		L3771
Dextran sulfate sodium salt	MP Biomedicals (Seoul, Republic of Korea)	02160110-CF

Table S2. Antibody information used in experiments.

Antibody	Source	Cat. No.
COX-2	Santa Cruz Biotechnology (Santa Cruz, CA, USA)	sc-376861
iNOS		sc-7271
STAT1		sc-592
p65		sc-372
β-actin		sc-81178
phospho-p65	Cell Signaling Technology (Danvers, MA, USA)	#3033
phospho-STAT1		#8826

Table S3. List of primers.

Gene		Sequence	Tm (℃)
TNF-α	Forward	AGCACAGAAAGCATGATCCG	55
	Reverse	CTGATGAGAGGGAGGCCATT	
IL-1β	Forward	ACCTGCTGGTGTGTGACGTT	55
	Reverse	TCGTTGCTTGGTTCTCCTTG	
IL-6	Forward	GAGGATACCACTCCCAACAGACC	55
	Reverse	AAGTGCATCATCGTTGTTCATACA	
β-actin	Forward	ATCACTATTGGCAACGAGCG	55
	Reverse	ATCACTATTGGCAACGAGCG	
